# Solitary extramedullary plasmacytoma in the lung misdiagnosed as lung cancer: A case report and literature review

**DOI:** 10.3389/fonc.2022.950383

**Published:** 2022-08-30

**Authors:** Jingjing Wang, Xiaoyun Yang, Xiaomei Liu, Tao He, Bin Liu, Lei Yang, Fei Yuan, Jing Li

**Affiliations:** ^1^ Department of Critical Care Medicine, Tianjin First Central Hospital, Tianjin, China; ^2^ Department of Radiology, Characteristic Medical Center of Chinese People’s Armed Police Force, Tianjin, China; ^3^ Department of Pathology, Characteristic Medical Center of Chinese People’s Armed Police Force, Tianjin, China; ^4^ Department of Pulmonary and Critical Care Medicine, Characteristic Medical Center of Chinese People’s Armed Police Force, Tianjin, China

**Keywords:** primary pulmonary plasmacytoma, extramedullary plasmacytoma, CT scan, pathology, prognosis

## Abstract

**Background:**

Extramedullary plasmacytoma (EMP) is an extremely rare kind of soft tissue plasma cell neoplasm without bone marrow involvement or other systemic characteristics of multiple myeloma. Primary pulmonary plasmacytoma (PPP), with no specific clinical manifestations, is an exceedingly rare type of EMP. Because of its complexity, PPP is often difficult to diagnose. Computed tomography-guided percutaneous core needle biopsy (CT-guided PCNB) has been shown to have high sensitivity, specificity and accuracy for characterization of pulmonary lesion, particularly if malignancy is suspected. Here we presented a rare case of PPP diagnosed with CT-guided PCNB.

**Case presentation:**

A 78-year-old female smoker who visited our outpatient clinic for a mass in the left lower lobe of the lung. Pathological based on CT-guided PCNB yielded a PPP with no lymph node or other distant metastasis.

**Conclusions:**

Extramedullary plasmacytoma should be considered in the differential diagnosis of a pulmonary mass.

## Background

Extramedullary plasmacytoma (EMP) is a rare monoclonal plasma cell tumor involving tissues outside the bone marrow. The entity comprises approximately 3–5% of all plasma cell neoplasms. More than 80% of EMP cases occur in the head and neck, and most cases involve the upper aerodigestive tract (1). Primary pulmonary plasmacytoma (PPP) is an extremely rare variant of EMP. In a comprehensive literature search reviewing patients with PPP, only 16 reports were found (2–15) summarized in **Table 1**). Here, we present an extremely unusual presentation as a pulmonary mass and without bone marrow involvement. With respect to the different biologic characteristics and prognoses of PPP, it is essential to consider in differential diagnosis of lung mass. CT-guided needle aspiration biopsy should be considered as first line to gain biopsy sample.

**Table 1:** Summary of the literature in the clinical treatment and prognosis of primary pulmonary plasmacytoma.

Author	Age	Gender	Radiography	Histopathology	Immunohischemistry	Immunofixation and/or	Bone marrow	Treatment	Prognosis
						eletrophoresis	biopsy		
Si Nie^2^	48	Male	A well-circumscribed	Large plasma cells	Positive for κ,	Not mentioned	Not mentioned	Chemotherapy	Disease free for
			mass in the left lower	with Russell bodies	CD138, CD38			(detail not	1.5 years after
			lobe dorsal segment		Negative for CD20,			mentioned)	surgery followed
					λ,CD79a				
Sang-Heon	26	Female	Infiltrative lesions in	Diffuse infiltration	Positive for λ	Serum electrophoresis:	Negative	Chemotherapy	Complete
Kim^3^			both lower lung lobes	of plasma cells		decreased albumin,			resolution after
						increased γ globulin			6 cycles therapy
Yi Zhou^4^	61	Female	A soft tissue mass in	Bronchial mucosa	Positive for LCA,CK,	Serum electrophoresis:	Negative	Surgery and	No recurrence for
					VIM,EMA,CD79a,	decreased albumin,		chemotherapy	1.5 years after
			the middle and lower	was infiltrated with	CD38,CD138	increased γ globulin		(melphalan and	surgery followed
			lobes of the right lung	inflammatory cells	Negative for CD3,	Serum immunofixation		prednisone)	
					CD68,S-100,κ,	electrophoresis:			
					λ,CD56	increased IgG,κ chain			
						and λ chain			
Rahim Y^5^	55	Male	A well-circumscribed	Infiltration by	Positive for MUM-1,	Serum immunofixation	Negative	Radiotherapy and	Tumor size
			opacity in the right	plasma cells with	CD138,CD56	showed IgG-λ		chemotherapy	reduced
			upper lung zone	moderate degree of		monoclonal		(bortezomib,	at 6 months
				nuclear atypia		gammopathy		cyclophosphamide,	
								dexamethasone)	
Maqsood	77	Female	A bilobed,	Medium sized atypical	Not mentioned	Not mentioned	Not mentioned	Radiotherapy	Not mentioned
U^6^			well-defined	plasmacytoid cells with					
			right apical mass	extracellular and					
				perivascular					
				amyloid deposition					
Zhang L^7^	92	Female	A mass detected in	Plasma cells with rich	Positive for CD38, CD56	Serum electrophoresis:	Not mentioned	Not mentioned	Not mentioned
			the right posterior	cytoplasm infiltrated in	CD56,VS38C,	decreased albumin,			
			thoracic cavity	lung tissue	CD138	increased γ globulin			
					Negative for CD3,				
					CD20,CD79a,				
					LCA,EMA				
Coelho	53	Male	Ovoid opacity in	Hypercellular light-	Positive for CD138, λ	protein electrophoresis	Negative	Radiotherapy	After 3 years
LRA^8^			the right hilar region	brownish fragments	Negative for CD3,	was normal			no finding of
				and well differentiated	CD20,κ				disease
				plasmacytoid cells with	AE1/AE3				recurrence
				small eccentric nuclei					
Z	60	Female	Bilateral alveolar	infiltration by	Positive for CD79a,	Serum electrophoresis:	Negative	Chemotherapy	After 4 monthly
Moham- mad			consolidation	plasmacytoid cells	CD138	M component		(melphalan and	courses chest
Taheri^9^				with fine chromatin	Negative for CD20, CK	in γ region		prednisolone)	X-ray became
									normal
Montero C^10^	59	Male	A tumor in the left	infiltration by	Positive for IgA-κ	protein electrophoresis	Negative	Surgical and	Disease free
			main bronchus and	plasmacytoid cells		was normal		radiotherapy	during a follow-up
			enlarged lymph nodes						of 10 years
	64	Male	A mass in the right	infiltration by	Positive for IgG,κ	protein electrophoresis:	Not mentioned	Radiotherapy	Disease free for
			upper lobe	plasmacytoid cells		increased IgG			15 years followed
	56	Male	A mass in right	infiltration by	Positive for IgA,κ	protein electrophoresis:	Negative	Radiotherapy and	Developed to
			upper lobe	plasmacytoid cells		increased IgA-κ		chemotherapy	septic shock
								(detail not	during
								mentioned)	3 cycle and died
Shi-Ping	42	Female	Right anterior	a solid mass made up	Positively for κ chains	plasma electrophoresis:	Negative	Surgery and	Symptoms
Luh^11^			mediastinal	mostly of plasma cells	Negatively for λ chains	Negative		chemotherapy	improved after
			shadow with multiple					(detail not	2 months
			pulmonary nodules					mentioned)	treatment
Nozomi	71	Female	A tumor in the right	monotonous medullary	Positively for IgG,λ,	Not mentioned	Not mentioned	Chemotherapy	After 3 courses
Niitsu^12^			middle lobe	proliferation of	CD79a,CD138,CD20			(melphalan,	therapy, mass
				mature plasma cells	Negatively for κ,CD3			prednisolne)	decreased in size
Geetha	79	Male	A right hilar mass	infiltration by	Postive for monoclonal	Not mentioned	Not mentioned	Right middle	Not mentioned
Joseph^13^				plasmacytoid cells	λ chains			lobectomy	
Takahiro	45	Female	Massive parenchymal	massive infiltration of	Positively for IgA,κ	Immunoelectrophoresis:	Negative	Chemotherapy	After 4 monthly
Horiuchi^14^			infiltrate in	lymphoidcells in		monoclonal IgA-κ		(melphalan and	courses, chest
			the lower lobes	interstitium		M-peak was recognized		prednisolone)	X-ray became
				and parenchyma		on electrophoresis			normal
James N	65	Male	A right hilar mass	metastatic small oval	Not mentioned	Serum electrophoresis:	Negative	A right upper	15 months without
Wise^15^				cells present with		increased M-protein		lobectomy	recurrence
				hyperchromatic nuclei					
				and occasional mitoses					

## Case presentation

**Figure 1:**
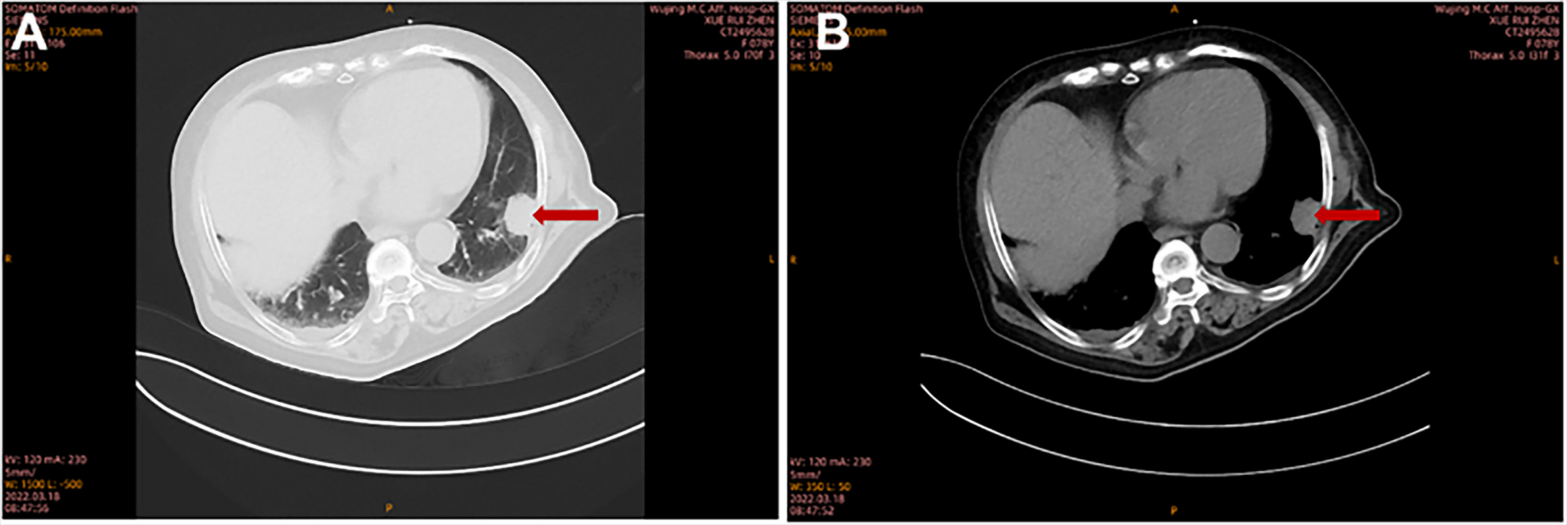
The imaging characteristics of PPP on pulmonary CT scan. Chest CT showed a solid lesion in the left lower lobe at lung window **(A)** and at mediastinal window **(B)**.

**Figure 2:**
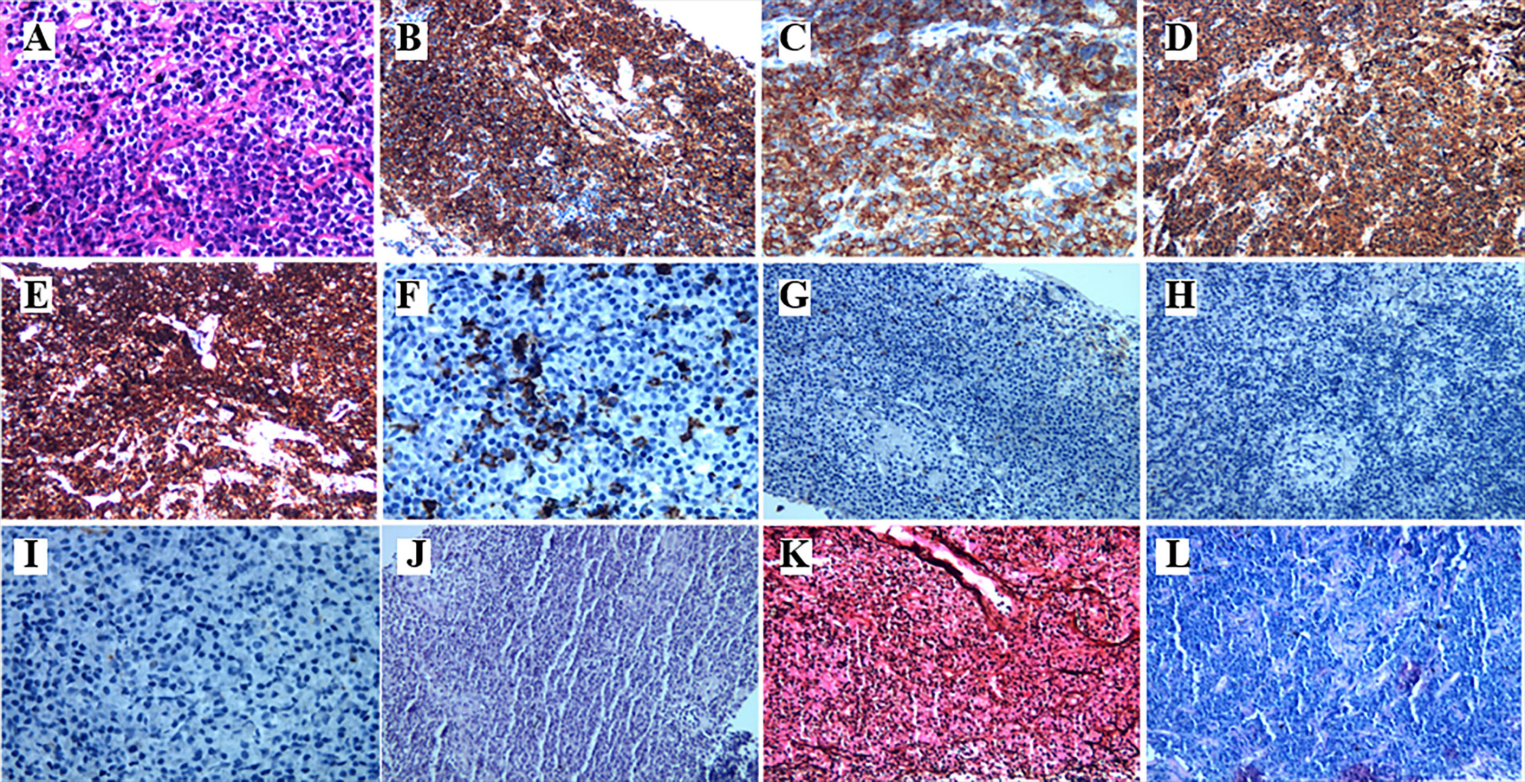
The histopathological characteristics of the tumor in the lung demonstrated by H&E and immunohistochemical staining. **(A)** Hematoxylin and eosin staining. The higher power view shows uniform small round blue cells with scant cytoplasm. **(B–I)** Immunohistochemical staining (400×magnification) was positive for CD38, CD138, Kappa, and LCA(CD45), CD20, but negative for CD56, CK, INSM1, respectively. **(J–L)** Specific stain of pathology of PAS, GMS, and acid fast stain, respectively.

**Figure 3:**
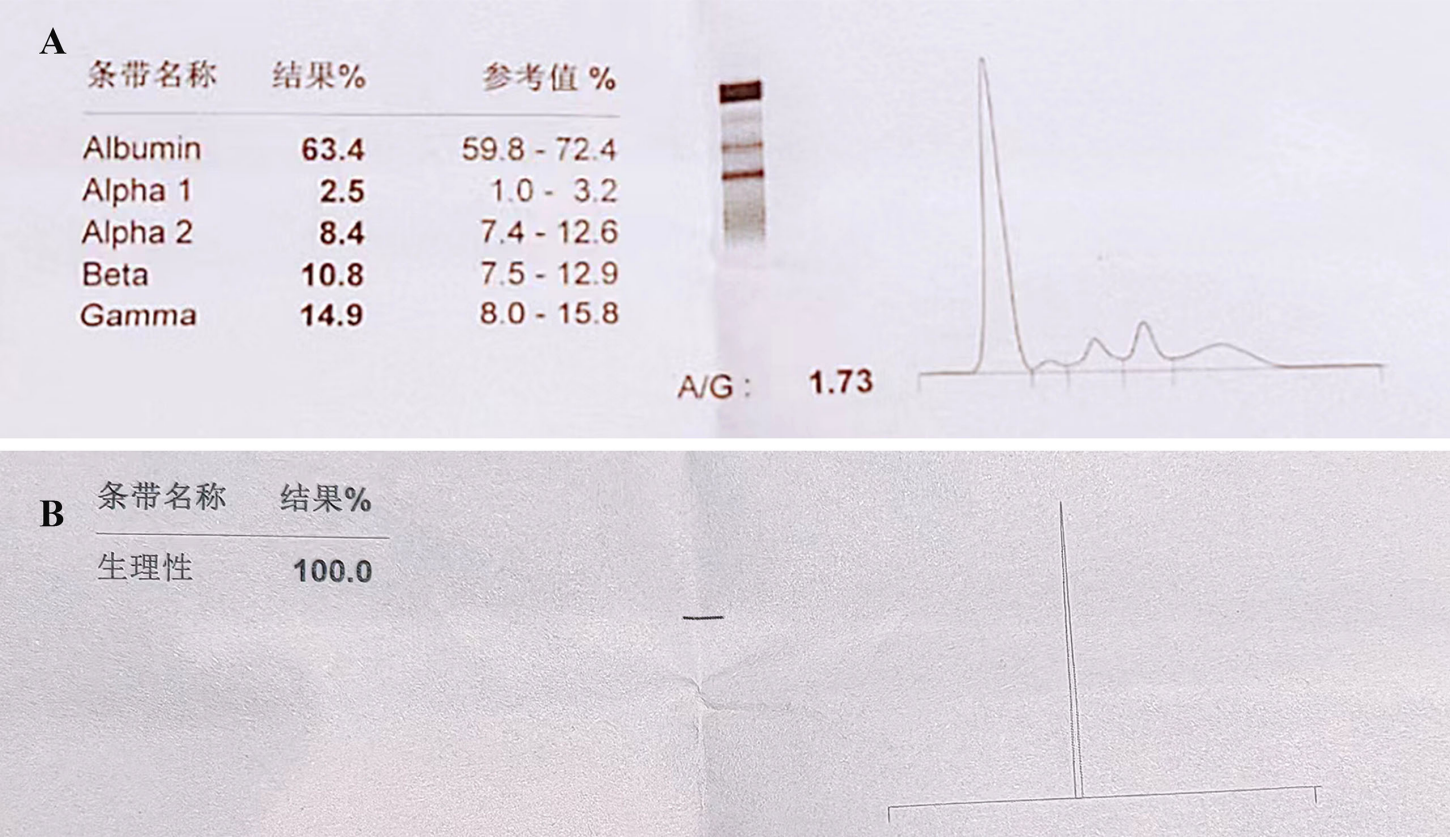
Serum and urine electrophoresis. No obvious abnormalities are detected in **(A)** Serum electrophoreis and **(B)** urine electrophoresis.

**Figure 4:**
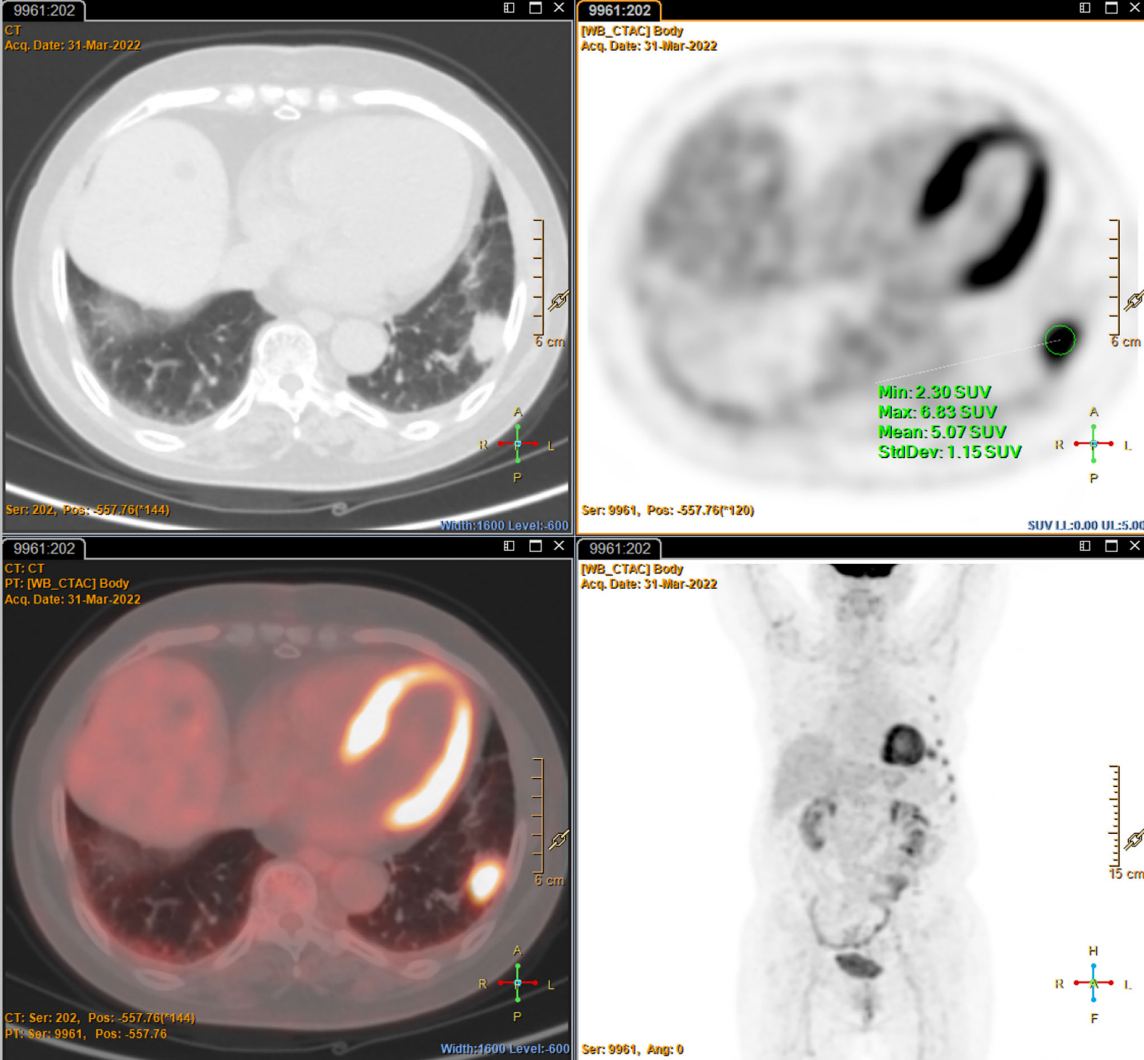
The Positron emission tomography-CT imaging characteristics shows a soft tissue density with hypermetabolism in the left lower lobe, but no abnormal metabolism in other organs and tissues, and no osteolytic lesions.

A 78-year-old female with a mass in the left lower lobe of the lung was referred to our hospital. The patient was diagnosed with hypertension and coronary heart disease prior. She had an approximately 60-year history of smoking. She has suffered from post-exercising dyspnea for 3 years without any apparent cause. The patient showed up with chest pain intermittent on the left, and the dyspnea was deteriorated a half months ago. No obvious abnormalities were found in physical examinations. A routine laboratory test showed white blood cell count, 4.77 ×10^9^/L; neutrophil percentage, 50.8%; haemoglobin, 122 g/L; blood platelet count, 152×10^9^/L; urine protein +/-. Serum calcium and phosphorus were within normal ranges. The rest of the routine laboratory examinations at the time of admission showed no obvious abnormalities. A chest computed tomography (CT) scan demonstrated a well-circumscribed mass measuring 23.14 × 21.33 × 20.28 mm located in left lower lobe (**Figure 1**). The mass was homogeneous and without any area of calcification or necrosis on a CT plain scan. It was marginal and spiculated with adjacent pleural retraction without bronchiolar obstruction. No obvious enlarged lymph nodes were found in the mediastinum. CT data resulted in a diagnosis of peripheral lung cancer. All the tumor marker was normal in the blood. Subsequently, the patient received CT-guided needle aspiration biopsy. The histological examination of the specimen revealed multiple lymphocyte and plasma cells were accumulated (**Figure 2A**). Immunohistochemistry was positive for LCA (CD45) (+++), CD 138 (+++), CD 38 (+++), kappa (+++), CK (-), CD 20(-), INSM1 (-), CD 56(-) (**Figures 2B–I**). In addition, specific stain of pathology including PAS, GMS were negative (**Figures 2J–L**). Pathological biopsy indicated extramedullary plasmacytoma. The patient was further evaluated by other diagnostic tests, including urine Bence-Jones protein, serum electrophoresis (**Figure 3A**), urine electrophoresis (**Figure 3B**), and bone marrow biopsy. However, all of the above tests had no abnormal change. The PET-CT revealed solid lesions with high grade increase 18F-fluorodeoxyglucose (18F-FDG) uptake in the lower left lobe of lung. There were no signs of abnormal metabolism in other organs and tissues, and no osteolytic lesions (**Figure 4**). Ultimately, the patient was diagnosed with PPP.

## Discussion and conclusions

In the present study, we encountered an extremely rare case of primary lung plasmacytomas without involving bone marrow. According to the classification of the Wilshaw method (4): Stage I, the tumour is confined to the primary site; stage II, the tumour has invaded local lymph nodes; and stage III, there are obvious widespread metastases. Therefore, in this case, the tumor should be classified as stage I.

Solitary plasmacytomas (SP) are rare neoplasms, involving solitary plasmacytoma of the bone (SPB), solitary extramedullary (extra-ossesous) plasmacytoma (SEP) or multiple solitary plasmacytomas (MSP) (16). Generally SP do not involve systemic manifestation or bone marrow. However, the entity has a propensity to eventually progress to MM (16). SEP is encountered more frequently in sites having a rich lymphatic drainage such as nasal cavity, nasopharynx, and upper respiratory tract (16).

In the present study, we encountered an unusual site of SEP. The average age of PPP was 60 years old, one fourth of patients were under 50 years old. No gender difference was showed. The symptoms of PPP were nonspecific and depended on the location of the neoplasms and their tumor classification. Most PPP presented solitary pulmonary masses, multiple shadowed masses was rare on CT image. Therefore, PPP is often misdiagnosed as tuberculosis or lung cancer. The imaging findings of PPP reported in the literature are mostly solitary pulmonary nodules or masses, mostly located around the hilar, with round or quasi-circular shape, relatively uniform density and clear boundary (2). A few patients also show multiple nodules, masses or diffuse lesions in the lung (3). Consistently with previous reports (2), the tumor in this case on CT image was rounded masses with well-defined margins that was initially misdiagnosed as peripheral lung cancer. There are some subtle differences between PPP and peripheral lung cancer, tuberculoma, but there is no characteristic difference. In general, peripheral lung cancer is deeply lobulated with short hard burrs. The radiography of tuberculoma shows no lobule with calcified foci, and satellite lesions around. Peripheral lung cancer shows obvious enhancement, but tuberculoma shows no or light strengthening on enhanced CT scan. PPP presents with moderate uniform reinforcement on enhanced CT scan (2). The tumor markers of pulmonary carcinoma can be elevated, while tumor marker was negative in tuberculoma and PPP. Consistent with the CT presentation of previous cases, the mass was homogeneous and necrosis and calcification are rarely seen in this case (17). Few patients showed diffuse consolidation in bilateral lung (3, 9). Castleman disease is also a lymphoproliferative disease with hyperplastic germinal center of the lymphatic node. According to the distribution of lymph nodes, it was divided into Unicentric Castlman disease and Multicentric Castlman disease. According to pathology, There are three pathologic subtype, including hyaline vascular type, plasma cell type and intermediate type (18). Unicentric Castleman’s disease, which mimic PPP, usually shows well-circumscribed and homogenous masses in mediatum with intense reinforcement on enhanced CT scan. Nevertheless, Unicentric Castleman’s disease predominantly consists of the hyaline vessel variant (90%) (19). Histopathology is the only method to make a definitive diagnosis. The feature of Castleman disease is multiple concentric rings of mantle zone lymphocytes encircling atretic follicles. However, CD 38 and CD 138 are indicative of the diagnosis of plasmacytoma, especially CD138 (2, 4). In most circumstances, features of PPP tumor cells are positive for CD138, CD38, CD45, PC, EMA, and CD20, while negative for CD15. In a few cases, CK and EMA are positive, but negative for CD45 (4).

The use of PET/CT in evaluation of plasma cell malignancy has opportunities of detecting MSP or additional bone lesions when biochemical and laboratory investigations are within normal limits (20). Eletrophoresis of most cases showed decreased albumin and increased γ globulin (3–5, 7) or M-peak (9, 14, 15). Consistent with few cases serum eletrophoresis showed normal (8, 10, 11). In this case, immunohistochemistry findings showed the infiltration of numerous lymphocyte and plasma cells positive for CD138 and CD38. PET-CT showed no evidence of osteolytic lesions and distant metastasis. Thus, a diagnosis of PPP was made.

There are no established treatments for patients with PPP. Anatomic pulmonary resection with or without radiotherapy are the most therapeutic approach for PPP (6). Adjuvant chemotherapy is usually conservative treatment in case of diffuse infiltration, aggressive lesion on histopathology, or poor local control after local surgery or radiotherapy (21, 22). Melphalan and prednisone are commonly used chemotherapy scheme (21, 22). Chemotherapy is considered in patients with tumors larger than 5cm (4). Taking into account, the size of the primary lesion without local infiltration or distant metastasis. We planned complete radiation therapy for the elderly patients with multiple comorbidities. Long-term survival in PPP remains unclear, due to limited follow-up data on too few patients. PPP has a relative favourable prognosis, as evidence by previous findings that these patients survived 10-20 years (10, 21). 20%-40% rate of progress to MM was noted (23). After 4 courses of radiotherapy, the chest X-ray became normal. Further follow-up is needed to monitor the progression of this case, and determine optimal treatment strategies for similar case.

In conclusion, we report an extremely rare presentation of SEP. This case highlights that attention should be paid to the differential diagnosis of pulmonary mass. Precise biopsy and optimal pathological evaluation will confirm the diagnosis. Doctors should be mindful of PPP as a differential diagnosis.

## Ethics statement

This case report was approved by the Medical Ethics Committee of Characteristic Medical Center of Chinese People’s Armed Police Force. The patient and her family consented to participate the study.

## Author contributions

The authors’ contributions are described as followed. JW and BL collected the data of medical history. JW wrote the manuscript. XY and XL collected the imaging data. TH collected the pathological data. LY and JL did the CT-guided needle aspiration biopsy. JL and FY revised the manuscript. JL and FY were the guarantors of this work and take responsibility for the contents of the article. All authors have contributed significantly and are in agreement with the content of the manuscript. All authors contributed to the article and approved the submitted version.

## Acknowledgments

The authors thank the patient for her participation and her agreement to the publication of the report. We thank Tianjin Key Medical Discipline (Specialty) Construction Project.

## Conflict of interest

The authors declare that the research was conducted in the absence of any commercial or financial relationships that could be construed as a potential conflict of interest.

## Publisher’s note

All claims expressed in this article are solely those of the authors and do not necessarily represent those of their affiliated organizations, or those of the publisher, the editors and the reviewers. Any product that may be evaluated in this article, or claim that may be made by its manufacturer, is not guaranteed or endorsed by the publisher.
